# Fire-Disturbed Soil and Intraspecific Competition Modulate Juvenile Performance of Invasive *Hakea decurrens* subsp. *physocarpa*

**DOI:** 10.3390/plants15203217

**Published:** 2026-10-20

**Authors:** Maria Cristina Morais, Helena Ferreira, João Ricardo Sousa, João Alexandre Cabral, Berta Gonçalves

**Affiliations:** Centre for the Research and Technology of Agroenvironmental and Biological Sciences, CITAB, Inov4Agro, Universidade de Trás-os-Montes e Alto Douro, UTAD, Quinta de Prados, 5000-801 Vila Real, Portugal; helenaf@utad.pt (H.F.); jricardo@utad.pt (J.R.S.); jcabral@utad.pt (J.A.C.); bertag@utad.pt (B.G.)

**Keywords:** invasive alien plant species, post-fire response, Mediterranean ecosystems, functional traits, seedling emergence

## Abstract

Fire has historically been an important component of Mediterranean ecosystems, where many invasive alien plant species (IAPS) spread by taking advantage of fire disturbance, out-competing native species and creating landscapes with novel fire regimes. In the serotinous woody IAPS *Hakea decurrens* subsp. *physocarpa* (hereafter *H. decurrens* for simplicity)*,* fire promotes fruit opening and subsequent recruitment, but the contribution of fire-disturbed soil and conspecific density to its establishment remains poorly understood. Thus, the present study evaluated the responses of juvenile *H. decurrens* to local natural soil (NS) and fire-disturbed soil (DS), in the absence (NC) or presence (WC) of intraspecific competition, during a 12-month outdoor experiment. A preliminary assay also characterized seedling emergence under different light conditions (full, moderate and low) to mimic the gap effect caused by fires. Seedling emergence ranged from 78.9% to 97.4%, with the lowest values observed under low light conditions. Soil condition did not affect relative height growth rate, whereas intraspecific competition significantly reduced growth rate and most final growth traits. Compared with NS, DS selectively altered shoot biomass, root development, proteoid root formation, leaf pigments, carbon metabolism and mineral composition, and was associated with higher easily extractable glomalin-related soil protein values, but did not consistently enhance overall plant growth. Soil × competition interactions were particularly evident for biomass allocation and carbohydrate metabolism. Principal component analysis revealed distinct functional profiles among treatment combinations. Overall, DS did not consistently enhance juvenile growth of *H. decurrens*. On the contrary, plants maintained performance through coordinated belowground, physiological, biochemical and nutritional adjustments, whereas intraspecific competition emerged as the strongest constraint on individual performance.

## 1. Introduction

Fire is a major ecological disturbance in Mediterranean ecosystems, with effects that extend from vegetation structure to soil functions [1]. Depending on fire intensity, duration and recurrence, soil properties and time since burning, forest fires may alter soil organic matter, nutrient availability, pH, microbial communities, and functional diversity [2,3] with effects ranging from transient nutrient enrichment to longer-term soil degradation [4]. These changes create a post-fire environment that can strongly influence vegetation recovery and plant establishment [5]. Fire reduces vegetation cover, modifies resource availability and alters competitive interactions [6,7], potentially creating conditions that may favor existing non-native species to colonize and invasive alien plant species (IAPS) to dominate [8]. The interaction between fire and IAPS is induced by a range of ecological mechanisms [9] that may differ depending on the IAPS, the composition of the community before fire, and the conditions under which fire occurs [7]. These mechanisms often result in positive feedback loops, where fire promotes the spread of IAPS, which in turn alter fuel properties and increase fire frequency or intensity, consequently suppressing native biodiversity and hindering ecosystem recovery [10]. Although post-fire invasion is commonly associated with propagule release, increased resource availability and reduced competition from resident vegetation, less is known about whether fire-altered soil conditions themselves enhance the subsequent performance of IAPS.

For newly established plants, post-fire soil conditions may represent a resource pulse or a nutritional constraint [11]. Ash deposition and combustion of organic matter can transiently alter nutrient availability, often resulting in net losses of organic matter and nitrogen [3], whereas subsequent erosion, leaching and nutrient uptake by regenerating vegetation may deplete soil resources [12]. Consequently, plant responses to burned soils are expected to depend strongly on species-specific strategies of resource acquisition and allocation [13]. This issue may be particularly relevant for Proteaceae, many of which form specialized cluster (proteoid) roots [14]. These structures increase soil exploitation and release compounds that mobilize poorly available nutrients, particularly phosphorus [14,15] and several micronutrients such as Fe, Zn and Mn [14]. The ability to adjust cluster root production may consequently buffer nutrient limitation and contribute to the persistence of proteaceous IAPS in post-fire environments, where soil fertility is often transiently or persistently reduced.

Another consequence of successful post-fire plant recruitment and recovery is the rapid increase in conspecific density [16]. Reduced competition from resident native vegetation may initially favor invasive species, but massive recruitment can subsequently intensify intraspecific competition for belowground resources, space and light. Density-dependent competition generally reduces individual plant size and can modify biomass allocation and resource-acquisition traits [17]. In invasion ecology, however, competitive studies have predominantly focused on interactions between invasive and native species [18,19], whereas the role of intraspecific competition in regulating invader performance remains comparatively less studied and appears strongly species dependent [20]. Because the intensity and outcome of intraspecific competition may vary with soil resource availability, post-fire soil condition and conspecific density may act jointly in determining juvenile performance.

*Hakea decurrens* subsp. *physocarpa* WR Barker (hereafter *H. decurrens* for simplicity) is an Australian Proteaceae IAPS that has become invasive in several regions outside its native range, including parts of Europe, New Zealand and South Africa, and has become particularly problematic in fire-prone Mediterranean-type environments such as France, New Zealand, Portugal, Spain and South Africa [21]. This IAPS was included in the European Union’s list of concern in 2022. In Portugal it was introduced for living fences and ornamental purposes [22] and it is considered an aggressive woody IAPS. It should be noted that invasive populations in Portugal were historically identified as *H. sericea*, but recent taxonomic evidence has shown that these populations actually correspond to *H. decurrens* subsp. *physocarpa* [23]. This IAPS can form dense, impenetrable, and sometimes extended mono-specific stands widely favored by anthropogenic disturbances [21], including fire events. *H. decurrens* possesses several traits that favor persistence in fire-prone environments, including a large canopy-stored seed bank (serotiny), heat-resistant fruits (follicles) that accumulate on a plant throughout its lifetime [24], and high seed survival and seedling recruitment after a fire [25]. Fire or death of the parent plant generally promotes follicle opening and the release of winged seeds [26,27], whose germination may extend over several months depending on environmental conditions [27]. In South Africa, estimated seed densities of up to 7500 seeds m^−2^ were found in the ash bed following fires [28]. Such massive recruitment can generate dense, even-aged cohorts [29], creating strong potential for subsequent intraspecific competition. Although regeneration is predominantly seed-based, resprouting can occasionally occur following pruning, particularly in smaller individuals [30]. In addition, the capacity of *Hakea* species to develop proteoid roots provides a potentially important mechanism for maintaining nutrient acquisition under altered or nutrient-poor soil conditions [25].

Despite the close association between fire and massive recruitment of *H. decurrens* [24,30], the mechanisms that determine subsequent juvenile establishment remain poorly understood. In particular, it is unknown whether fire-disturbed soil directly enhances plant performance or whether post-fire success instead reflects the ability of the species to maintain growth through functional adjustment to altered soil conditions. Furthermore, the extent to which increasing conspecific density modifies these responses has received little attention.

We therefore evaluated the individual and interactive effects of soil condition and intraspecific competition on juvenile growth of *H. decurrens*, biomass allocation, leaf biochemical responses and mineral composition during a 12-month outdoor experiment. A preliminary emergence assay was additionally used to characterize seedling emergence across contrasting soil and light conditions. We hypothesized that: (H1) fire-disturbed soil would enhance overall juvenile growth of *H. decurrens*, (H2) intraspecific competition would reduce individual performance of *H. decurrens*, and (H3) soil condition and intraspecific competition would jointly influence plant functional responses, resulting in a significant soil x intraspecific competition interaction. By jointly considering the effects of soil condition and conspecific density, this study aimed to determine whether post-fire establishment and dominance of *H. decurrens* is driven by fire-disturbed soil or by its intrinsic capacity for functional adjustment, with implications for post-fire management strategies in invaded Mediterranean ecosystems.

## 2. Materials and Methods

### 2.1. Collection of Fruits of Hakea decurrens subsp. physocarpa and Soil

Fruits (follicles) of *Hakea decurrens* subsp. *physocarpa* (hereafter simplified to *H. decurrens*) were collected from several adult plants at the end of 2017 across the Marão mountain range, at elevations of 400–800 m a.s.l. (approximate central coordinates: 41.309° N, 7.974° W). The area is located within the Alvão/Marão Natura 2000 site (PTCON0003, 41.39 °N, −7.83 °W), within the Mediterranean biogeographical region. This Site of Community Importance covers approximately 58,788 ha and comprises a variety of natural and semi-natural habitats dominated by *Quercus robur*, *Quercus pyrenaica*, *Erica* spp. and *Ulex* spp., together with extensive *Pinus pinaster* plantations. Soils in the area are predominantly classified as Cambisols and Leptosols developed over schist and granite.

*H. decurrens* was introduced into the Marão mountain range as a hedge plant at the beginning of the 20th century, and subsequently expanded, particularly following the 2013 wildfire. Currently, the species occupies different elevational zones, especially those previously dominated by *P. pinaster*, where dense, continuous stands are found. Fruits were transported to the laboratory in paper bags. Under laboratory conditions, the fruits were air dried to promote follicle opening and seed release. Seeds were then stored in paper bags at room temperature until use.

**Figure 1:**
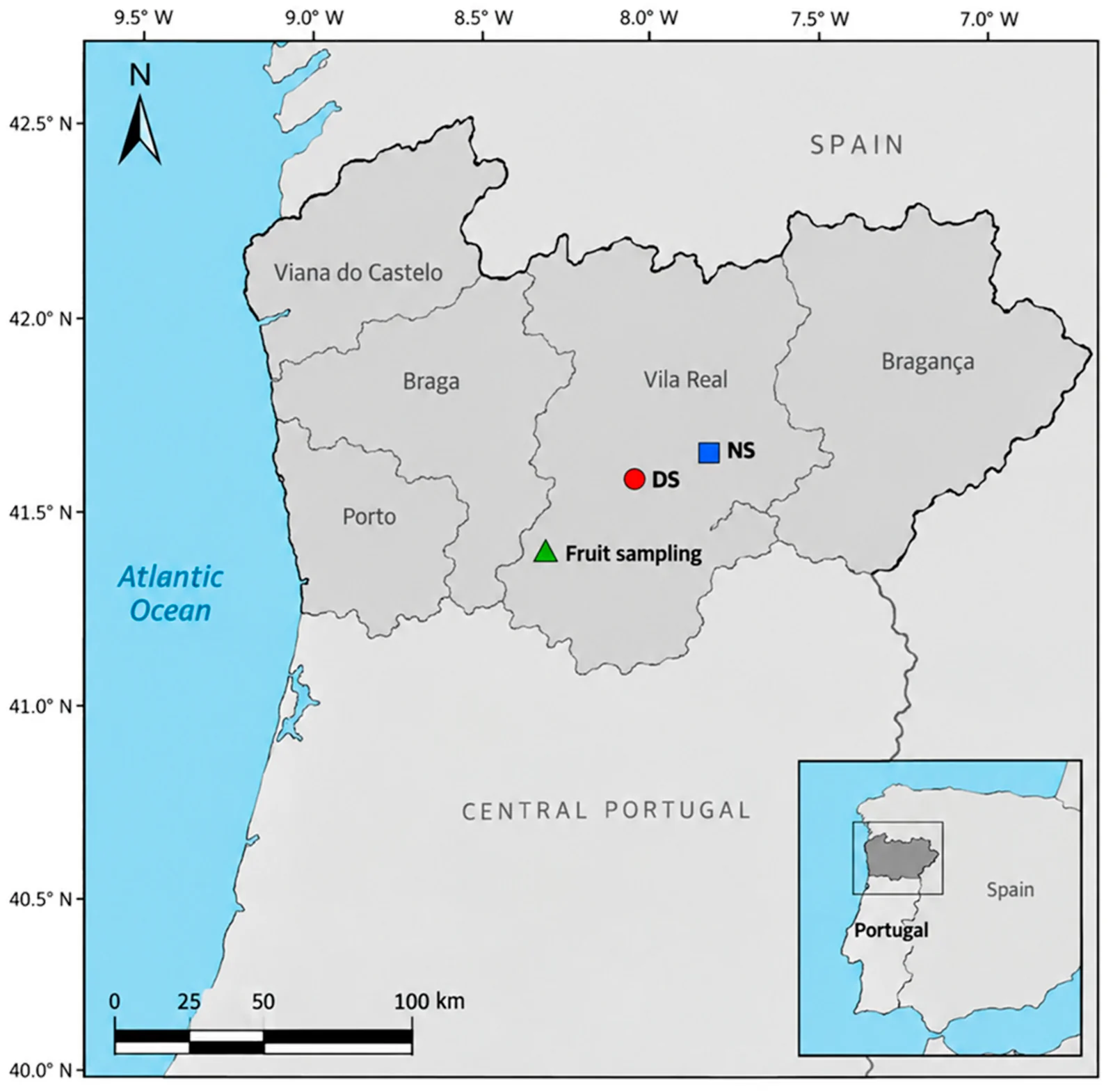
Geographic location of the sampling sites used in this study, including the fruit collection site, the local natural soil (NS) site and the fire-disturbed soil (DS) site.

The upper 0–10 cm soil layer was collected in September 2018 from two locations near the Alvão/Marão region, in the Alto Tâmega sub-region (NW Portugal), characterized by Cambisols developed on granitic parent material. Local natural soil (NS) was obtained from an area with an intact native plant community, dominated by *P. pinaster* plantations, located near Vila Pouca de Aguiar (41.528° N, 7.632° W, 730 m a.s.l.), whereas fire-disturbed soil (DS) was collected at Vilarinho, Ribeira de Pena (41.501°N, 7.822° W, 700 m a.s.l.) from an area affected by a severe wildfire in August 2017. The geographic location of the sampling sites is shown in Figure 1.

All the areas lie in a transitional zone between Atlantic and Mediterranean climatic influences. As a regional climatic reference, the 1981–2010 climatological normal for the Vila Real Meteorological Station (41.2742 N, 7.7171 W; 561.5 m a.s.l.) indicates a mean annual air temperature of 13.6 °C, with monthly mean temperatures ranging from 6.0 °C in January to 21.7 °C in August. Mean annual precipitation is 1018.0 mm, concentrated mainly during autumn and winter, whereas summer is markedly drier, with mean monthly precipitation decreasing to 12.8 mm in July and 24.4 mm in August [31].

A total of 10 samples were collected at each location and pooled to obtain one composite sample per soil condition. The pooled soils were sieved (2 mm mesh) to remove seeds, coarse roots and organic debris, and stored at room temperature until used in the emergence and growth experiments. A representative subsample of each soil condition was kept refrigerated for physicochemical characterization at the Soil and Plant Laboratory of Universidade de Trás-os-Montes e Alto Douro (UTAD) following standard protocols. Soil pH was determined in a 1:5 (*w*/*v*) soil solution ratio using H_2_O and 1 mol L^−1^ KCl [32], and electrical conductivity in a 1:5 (*w*/*v*) soil to deionized water suspension [33]. Soil organic matter was determined by loss on ignition at 550 °C for 4 h in a muffle furnace [34], and total N was quantified by the Kjeldahl method [35]. Soil organic carbon was estimated from soil organic matter, assuming that organic carbon accounts for 58% of soil organic matter [36]. Effective cation exchange capacity (CEC), calculated from the sum of exchangeable Ca, Mg, K, and Na plus exchangeable acidity, was determined after extraction with barium chloride and analyzed by ICP-OES [37]. Extractable P and K were determined using the Egner–Riehm extraction method [38]. Extractable Cu, Zn, Fe, and Mn were obtained using a 1:10 soil-to-AAAc-EDTA solution at pH 4.65 [39] and quantified by atomic absorption spectroscopy, while extractable B was determined in hot water (1:2 soil-to-water ratio) and analyzed by ICP-OES [40]. The main physicochemical properties of both soils are presented in Table 1.

### 2.2. Seedling Emergence Assay

Seeds of *H. decurrens* were surface sterilized by first soaking them in 96% ethanol for 30 s and then in 4% commercial bleach for 2 min, followed by six rinses with distilled water.

Soil from each condition (NS and DS) was used to fill 56-cell plastic trays (45.5 × 30.0 × 10.5 cm). Each tray was divided into three sections, corresponding to three seed densities (1, 2, 3 seeds/cell), with 18 cells assigned to each density. Three trays were prepared for each soil condition and exposed to one of the three light intensity levels: 100% (full light, FL), 50% (moderate light, ML) and 25% (low light, LL) of maximum light intensity (250 µmolm^−2^s^−1^), selected to mimic different canopy-gap conditions. Light intensity was measured with a portable radiometer/photometer (Macam Q102, Macam Photometrics Lda., Livingston, Scotland). Trays were placed in a growth chamber (Fitoclima 10000 EHHF, ARALAB, Sintra, Portugal) under controlled conditions: 12 h light (25 °C)/12 h dark (18 °C) and 60% relative humidity. All trays were watered every two days and their position within the growth chamber was randomly changed weekly to minimize positional effects.

Seedling emerged was recorded every two days for 90 days. Seeds were considered emerged when the seedling overcame the topsoil and became visible. Emergence-related parameters were determined per soil and light conditions (Table 2), including the emergence percentage (G, %), mean emergence time (MGT, day), coefficient of velocity of emergence (CVG, %), time to 50% emergence (T50, day), and synchrony of emergence (Z, without units) were determined, adapting the formulas described by [41,42].

### 2.3. Outdoors Experiment

After six months of growth in the growth chamber, the trays were transferred outdoors (maintaining the light conditions) for a 90-day acclimatization period while maintaining the corresponding light treatments and watering every two days. Plants exposed to 25% light intensity and those originating from the highest seed density (3 seeds/cell) were not included in the subsequent experiment because the number of healthy surviving plants was not sufficient to establish the planned experimental design. Plants from the high and moderate light intensities (100% or 50%) exhibited similar morphological characteristics, and the experiment was then arranged in a 2 × 2 factorial design, comprising two soil conditions (NS and DS) and two levels of intraspecific competition: one plant per pot (NC) and two plants per pot (WC). Therefore, eight individual healthy plants (NC treatment) and eight pairs of plants (WC treatment) of uniform height were chosen from each soil condition (NS and DS), and were transferred for 6 L pots filled with the same soil used in the emergence assay. Plants were allowed to grow outdoors under natural conditions, for 12 months. Pots were watered every two days with tap water, randomly arranged, and rotated weekly to minimize positional effects.

#### 2.3.1. Growth Measurements

Plant height, defined as the maximum shoot length, was measured at the beginning of the outdoor experiment (0 months) and subsequently at 3, 6, 9, and 12 months (harvest). At harvest, six pots per treatment combination were used for growth and biomass allocation measurements. The following measurements were performed: (i) maximum shoot and root length; (ii) shoot and root dry weight, obtained after oven-drying at 60 °C for 48 h until a constant dry weight; and (iii) root mass fraction (RMF), calculated as root biomass divided by the total plant dry biomass [43]. Basal stem diameter, number of branches and number of proteoid roots were also recorded. The plant-height relative growth rate (RGR_H_, i.e., the rate of height increases per height unit, per month) was calculated for the intervals 0–3, 3–6, 6–9, and 9–12 months using the formula: RGR_H_ = (ln H2 − ln H1)/(t2 − t1), where H1 and H2 are the plant height at the beginning and end of each interval, respectively, and t1 and t2 are the corresponding measurement times in months.

After growth measurements, a subsample of leaves was immediately frozen in liquid nitrogen and stored at −80 °C for biochemical analyses (photosynthetic pigments and carbohydrates). The remaining leaf material was subsequently oven-dried at 60 °C and ground to a fine powder for mineral analysis.

#### 2.3.2. Leaf Biochemical Determinations

Leaf material for biochemical analyses was collected at harvest, 12 months after the beginning of the outdoor experiment. Chlorophyll a [chl a], chlorophyll b [chl b] and total chlorophyll [chl total] were quantified in 80% cold acetone (*v*/*v*), according to Sesták et al. [44]. Total carotenoid [carot] contents were also extracted in 80% cold acetone (*v*/*v*) following the protocol of Lichtenthaler [45]. Total phenolic content was determined using the Folin–Ciocalteu method [46], in which absorbance of the sample solution was measured at 725 nm and phenolic content was quantified using a calibration curve prepared with gallic acid. Results were expressed as mg gallic acid equivalents per gram of fresh weight (mg GAE g^−1^ FW). Total soluble sugars (SS) and starch concentration were determined using the anthrone method, with D-glucose as a standard. SS were extracted by heating the samples in 80% ethanol for 1 h at 80 °C, and the methodology proposed by Irigoyen et al. [47] was adopted for quantification. Starch was extracted from the same solid fraction by heating the samples in 30% perchloric acid for 1 h at 60 °C, according to the protocol of Osaki et al. [48]. Absorbance measurements were performed using a SPECTROstar Nano Microplate Reader (BMG LABTECH, Ortenberg, Germany).

#### 2.3.3. Leaf Mineral Determinations

Dried leaf material was analyzed for macro and micronutrients (P, K, Ca, Mg, S, B, Fe, Cu, Zn, and Mn) after acid digestion using a HNO_3_ and H_2_O_2_ mixture, and element concentrations were quantified by inductively coupled plasma optical emission spectrometry (ICP-OES). All analyses were performed at the Soil and Plant Laboratory of UTAD.

#### 2.3.4. Easily Extractable Soil Glomalin Determination

At the end of the outdoor experiment, a sample of soil from each pot was collected and storage under refrigerated conditions until its analysis. Easily extractable glomalin (EEG) was determined using a modified protocol [49] based on the Bradford protein assay [50]. The EEG fraction was extracted from 0.25 g of 0.20 mm sieved soil using 2 mL of 20 mM sodium citrate (pH 7.0) at 121 °C for 60 min in an autoclave. After extraction, soil particles were removed by centrifugation at 3000× *g* for 15 min (21 °C). The EEG contents were quantified spectrophotometrically at 595 nm using a SPECTROstar Nano Microplate Reader (BMG LABTECH, Ortenberg, Germany) against a standard curve with varying concentrations of bovine serum albumin.

### 2.4. Statistical Analysis

Data on seedling emergence were interpreted descriptively, and no inferential statistical comparisons were performed because the experiment was conducted without independent experimental replication.

Data of plant growth are presented as the mean ± SE (standard error) of six independent pots from each treatment combination (n = 6). For pots containing two plants, individual plant measurements were averaged within each pot, and the pot mean was used as the experimental unit.

The effects of soil condition (local natural soil, NS; fire-disturbed soil, DS), intraspecific competition (1 plant per pot, NC; 2 plants per pot, WC) and their interaction were examined using a two-way analysis of variance (ANOVA). Each response variable was analyzed separately as an a priori-defined biological endpoint. When a significant soil × competition interaction was detected, differences among treatment combinations were examined using Tukey’s HSD post hoc test at a 95% confidence level, thereby controlling the risk of Type I error rate for multiple pairwise comparisons within each response variable. The assumptions of homogeneity of variances and normality of residuals were assessed using Levene and Shapiro–Wilk tests, respectively, and no significant violations were detected.

The plant-height relative growth rate (RGR_H_) was analyzed using a repeated-measures ANOVA, with growth interval (0–3, 3–6, 6–9 and 9–12 months after transplantation) as the within-subject factor and soil condition and intraspecific competition as between-subject factors. Sphericity was assessed using Mauchly’s test, and pairwise comparisons among growth intervals were adjusted using the Bonferroni correction.

A heatmap was used to present the relationship between each soil x competition treatment combination (NS-NC, NS-WC, DS-NC and DS-WC) in leaf mineral composition. Each mineral was standardized using z-scores calculated from individual experimental units, and mean z-scores were subsequently obtained for each soil × competition combination. Positive and negative z-scores indicate concentrations above and below the overall mean for each mineral, respectively.

Principal component analysis (PCA) was performed to determine which traits explain most of the variation under each treatment. Nine traits were included in the final PCA after excluding highly redundant variables. To avoid the influence of the unit of each parameter on the result, all data were standardized. Sampling adequacy was evaluated using the Kaiser–Meyer–Olkin (KMO) measure and Bartlett’s test of sphericity.

All statistical analyses were performed using the software package IBM SPSS version 29 for Windows (New Orchard Road, Armonk, NY, USA).

## 3. Results

### 3.1. Effects of Soil and Light Conditions on Seedling Emergence Parameters of H. decurrens

Seedling emergence of *H. decurrens* showed high values across all experimental conditions, ranging from 78.9% to 97.4%. (Table 3). The lower emergence values were observed under low-light conditions in both NS and DS (78.9% and 79.8%, respectively), whereas germination exceeded 89% under full and medium light. MGT, CVG and T50 showed relatively small variation among treatments, while emergence synchrony was numerically higher under full light than under moderate or low light.

### 3.2. Effects of Soil and Intraspecific Competition on Growth Parameters of H. decurrens

At the end of the 12-month experiment, significant soil × competition interactions were detected for shoot dry biomass (F_1,20_ = 5.063, *p* = 0.036) and RMF (F_1,20_ = 7.921, *p* = 0.011) (Table 4), indicating that the effects of soil condition and intraspecific competition on these traits were not independent. Post hoc comparisons showed that shoot dry biomass was highest in NS-NC plants and lowest in plants grown under competition in both soil conditions. The reduction associated with competition was more pronounced in NS, and differences between soil conditions were evident in the absence, but not in the presence, of competition. For RMF, competition had little effect in NS, whereas plants grown in DS showed a marked decrease in RMF under competition. For the remaining growth traits, no significant soil × competition interactions were detected, allowing the main effects of soil condition and competition to be interpreted independently.

Intraspecific competition significantly affected most growth parameters of *H. decurrens* (Table 4). Plants grown under competition exhibited lower height (F_1,20_ = 7.071, *p* = 0.015), shoot (F_1,20_ = 49.932, *p* < 0.001) and root dry biomass (F_1,20_ = 33.672, *p* < 0.001), root length (F_1,20_ = 21.972, *p* < 0.001), stem diameter (F_1,20_ = 24.575, *p* < 0.001) and number of proteoid roots (F_1,20_ = 31.699, *p* < 0.001). Competition also affected RMF (F_1,20_ = 6.948, *p* = 0.016), whereas branch number was unaffected (F_1,20_ = 3.953, *p* > 0.05).

Soil condition had a more selective influence on plant growth. Shoot dry biomass (F_1,20_ = 5.277, *p* = 0.033), root length (F_1,20_ = 4.504, *p* = 0.047) and proteoid root number (F_1,20_ = 4.721, *p* = 0.043) differed between NS and DS, whereas height, root biomass, stem diameter, branch number and RMF were not significantly affected by soil condition. Plants grown in DS showed numerically greater root length and a higher number of proteoid roots than those grown in NS.

**Figure 2:**
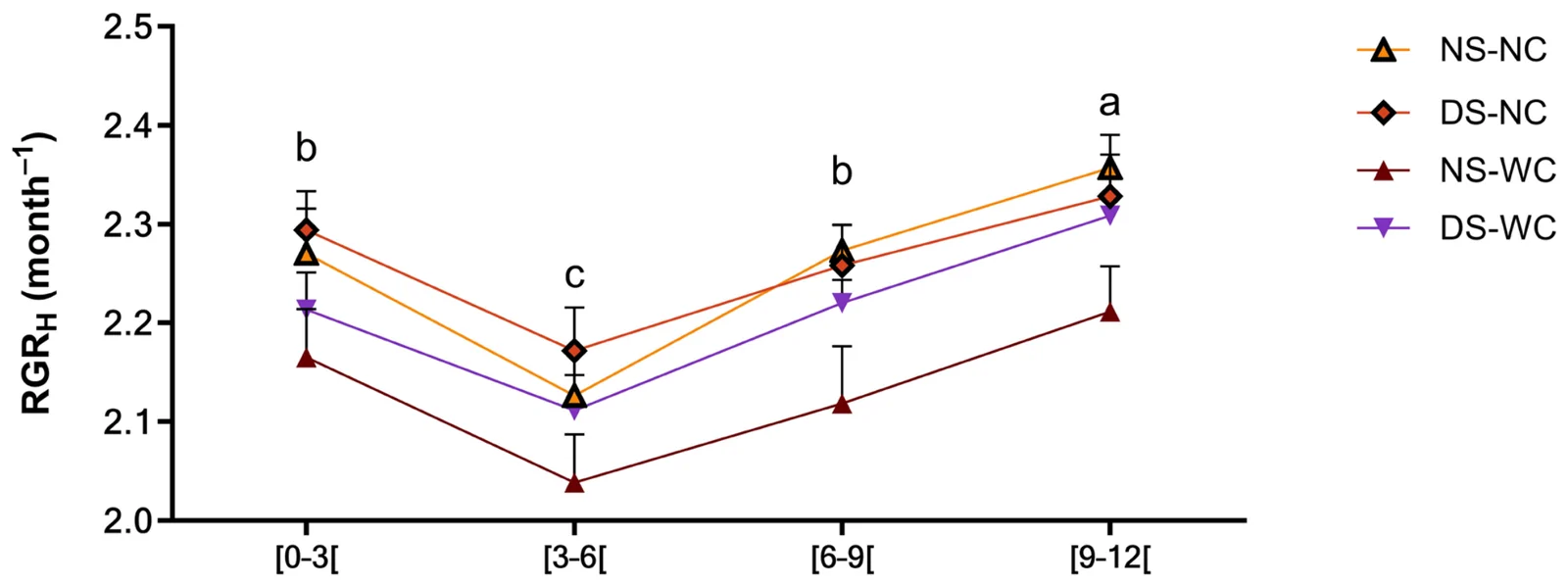
Variation of mean plant-height relative growth rate (RGR_H_) of *H. decurrens* plants grown in local natural soil (NS) and fire-disturbed soil (DS), without (NC) or with (WC) intraspecific competition, at the intervals [0–3[, [3–6[, [6–9[, and [9–12[ months. Values are means ± SE (n = 6). Different lowercase letters indicate significant differences among time intervals following the Bonferroni correction (*p* < 0.05).

RGR_H_ showed a marked temporal variation (F_3,57_ = 49.547, *p* < 0.001), reaching its lowest values during months 3–6 and its highest values during months 9–12 (Figure 2). RGR_H_ during months 0–3 and 6–9 did not differ significantly from each other. In addition, RGR_H_ was significantly affected by intraspecific competition (F_1,19_ = 5.882, *p* = 0.025), with plants grown under competition exhibiting lower RGR_H_ than plants grown individually. On the contrary, soil condition had no significant effect on the parameter (F_1,19_ = 1.383, *p* = 0.254). No significant time × soil (F_3,57_ = 0.259, *p* = 0.854), time × competition (F_3,57_ = 0.227, *p* = 0.877), or time × soil × competition interactions (F_3,57_ = 1.511, *p* = 0.221) were detected. The between-subject soil × competition interaction was also not significant (F_1,19_ = 1.031, *p* = 0.323).

### 3.3. Effects of Soil and Intraspecific Competition on Leaf Biochemical Parameters of H. decurrens

Significant soil × competition interactions were detected for chl b (F_1,20_ = 4.530, *p* = 0.046), the carotenoid-to-total chlorophyll ratio (F_1,20_ = 14.323, *p* = 0.001), SS (F_1,20_ = 9.605, *p* = 0.006) and starch (F_1,20_ = 45.733, *p* < 0.001) (Table 4). For chl b, concentrations were higher in plants grown in DS than in NS under both competition levels, although the magnitude of the soil effect differed according to competition. The carotenoid-to-chlorophyll ratio was highest in WC plants grown in NS, whereas SS concentration was highest in NC plants in NS. Starch showed a contrasting response to competition between soil conditions: competition increased starch concentration in NS but decreased it in DS. For the remaining biochemical traits, no significant soil × competition interactions were detected.

Plants grown in DS showed higher chl a (F_1,20_ = 96.645, *p* < 0.001), chl b (F_1,20_ = 82.893, *p* < 0.001), total chlorophyll (F_1,20_ = 93.363, *p* < 0.001) and carotenoid (F_1,20_ = 53.005, *p* < 0.001) concentrations than those grown in NS. Intraspecific competition had no significant effect on chl a, chl b or total chlorophyll, but significantly increased the chl a/chl b ratio (F_1,20_ = 8.395, *p* = 0.009) and reduced carotenoid concentration (F_1,20_ = 17.793, *p* < 0.001). Phenolic concentration increased significantly in both DS (F_1,20_ = 15.388, *p* < 0.001) and under WC (F_1,20_ = 31.669, *p* < 0.001).

### 3.4. Effects of Soil and Intraspecific Competition on Leaf Mineral Content of H. decurrens

A significant soil × competition interaction was only observed for Zn (F_1,20_ = 4.968, *p* = 0.038), with the highest concentration occurring in DS-WC plants (Table 4).

Leaf macronutrient concentrations were largely unaffected by soil condition and competition, with no significant effects detected for P, Ca, Mg or S (Table 4). Intraspecific competition significantly reduced leaf K concentration (F_1,20_ = 6.401, *p* = 0.020). In contrast, several micronutrients responded to soil condition. Leaf B and Cu concentrations were significantly higher in DS than in NS (F_1,20_ = 7.579, *p* = 0.012 and F_1,20_ = 7.711, *p* = 0.012 for B and Cu, respectively), whereas Mn concentration was lower in DS (F_1,20_ = 11.060, *p* = 0.003). Iron concentration increased significantly both in DS (F_1,20_ = 50.973, *p* < 0.001) and under competition (F_1,20_ = 22.030, *p* < 0.001), while Mn also increased under competition (F_1,20_ = 11.310, *p* = 0.003).

**Figure 3:**
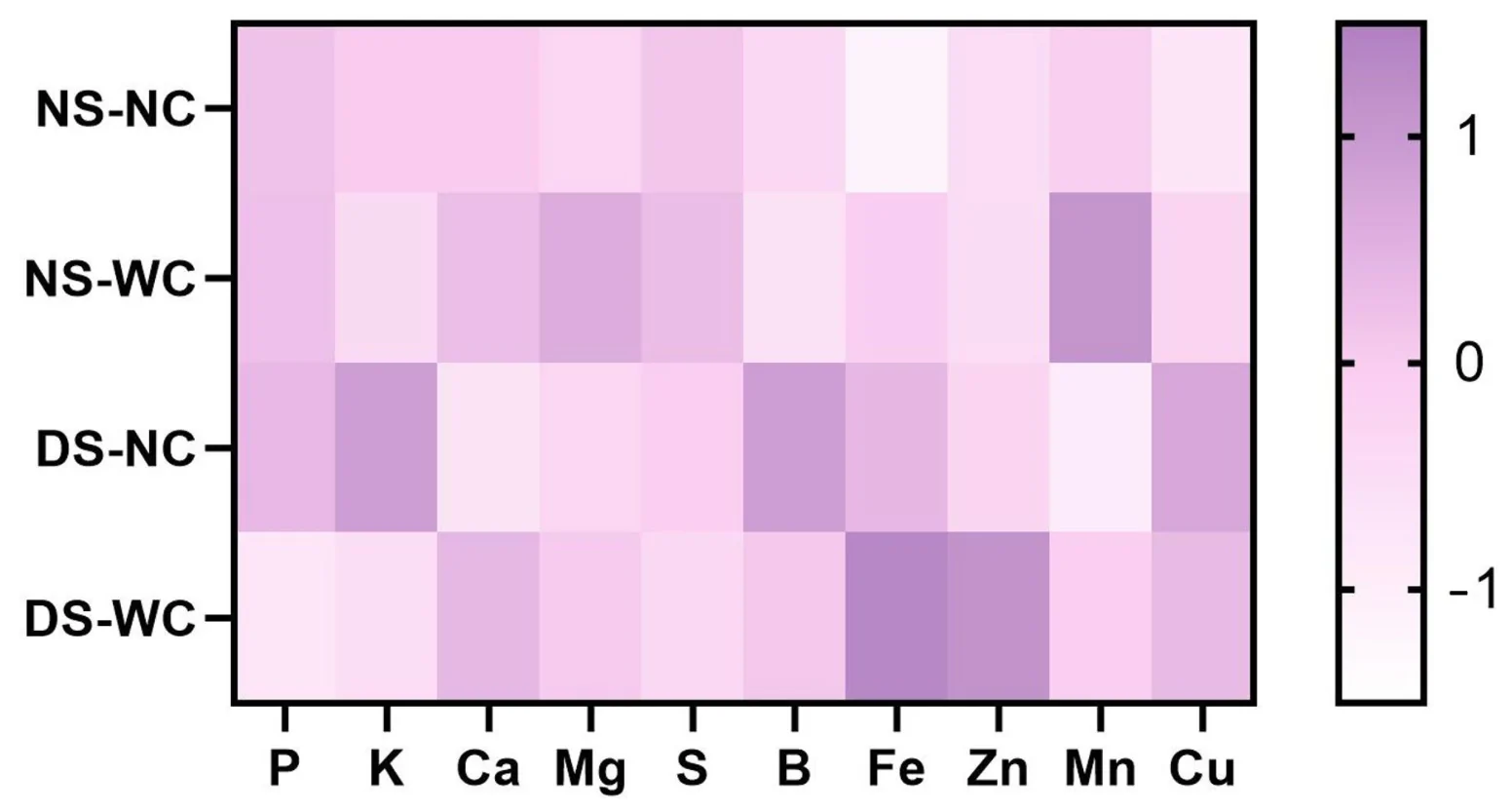
Heatmap of standardized leaf mineral concentrations in *H. decurrens* plants grown under each soil x competition treatment combination: local natural soil (NS) and fire-disturbed soil (DS), without (NC) or with (WC) intraspecific competition. The color scale indicates mean Z-scores for macronutrients (P, K, Ca, Mg, S) and micronutrients (B, Fe, Zn, Mn, Cu) in the different treatment combinations.

Overall, DS induced a selective shift in leaf mineral composition, particularly affecting micronutrients. In contrast, intraspecific competition further modified the leaf mineral profile, particularly K, Fe and Mn, while Zn exhibited a soil-dependent response to competition (Figure 3).

### 3.5. Effects of Soil and Intraspecific Competition on Easily Extractable Soil Glomalin

**Figure 4:**
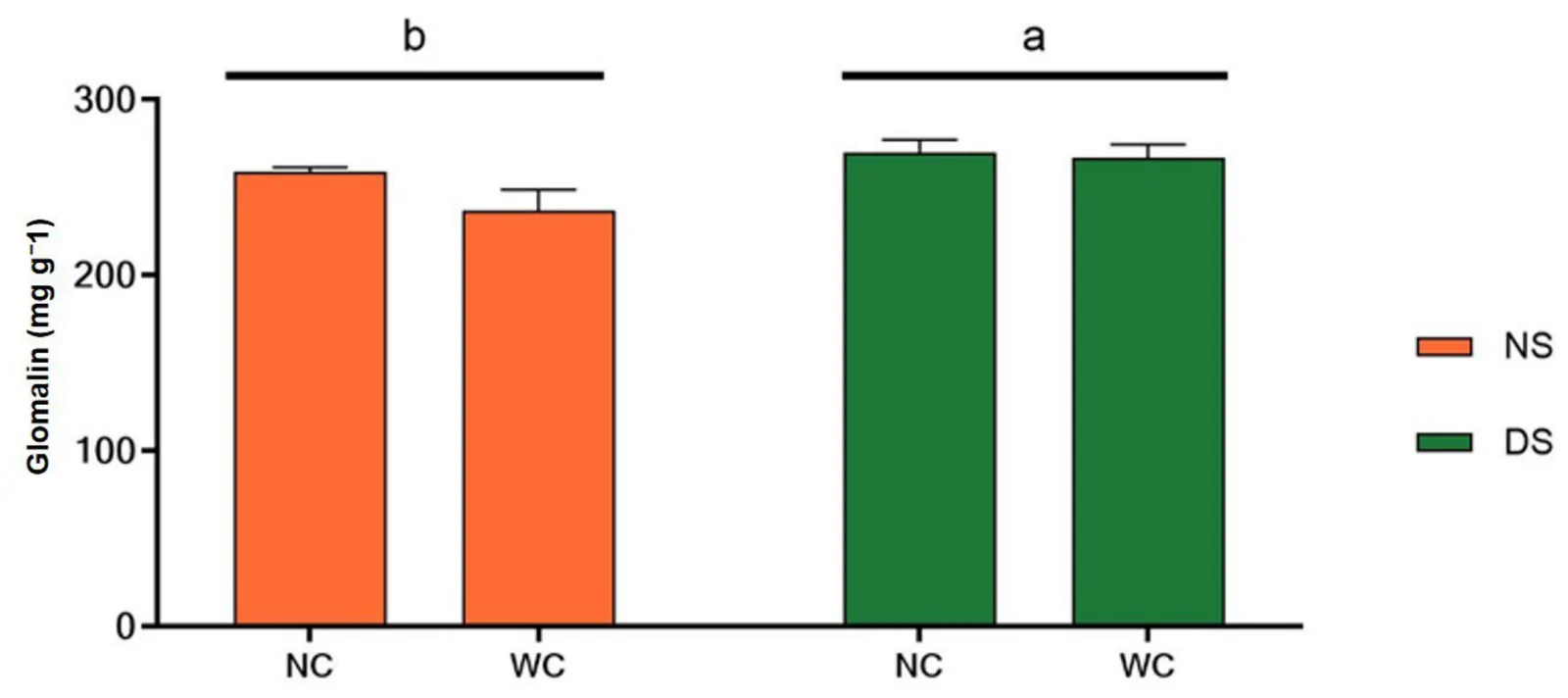
Easily extractable glomalin (EEG) determined in local natural soil (NS) and fire-disturbed soil (DS), without (NC) or with (WC) intraspecific competition, at the end of the 12-month experiment. Values are means ± SE (n = 6). Different lowercase letters indicate significant differences among soil × competition combinations following the Tukey test post hoc multiple comparisons (*p* < 0.05).

EEG concentration varied between 236 ± 12 to 269 ± 8 mg g^−1^ (Figure 4). It was significantly affected by soil condition (F_1,20_ = 6.326, *p* = 0.021), with higher values in DS than in NS, whereas neither intraspecific competition (F_1,20_ = 2.289, *p* > 0.05) nor the soil × competition interaction had a significant effect (F_1,20_ = 1.413, *p* > 0.05).

### 3.6. Principal Component Analysis

**Figure 5:**
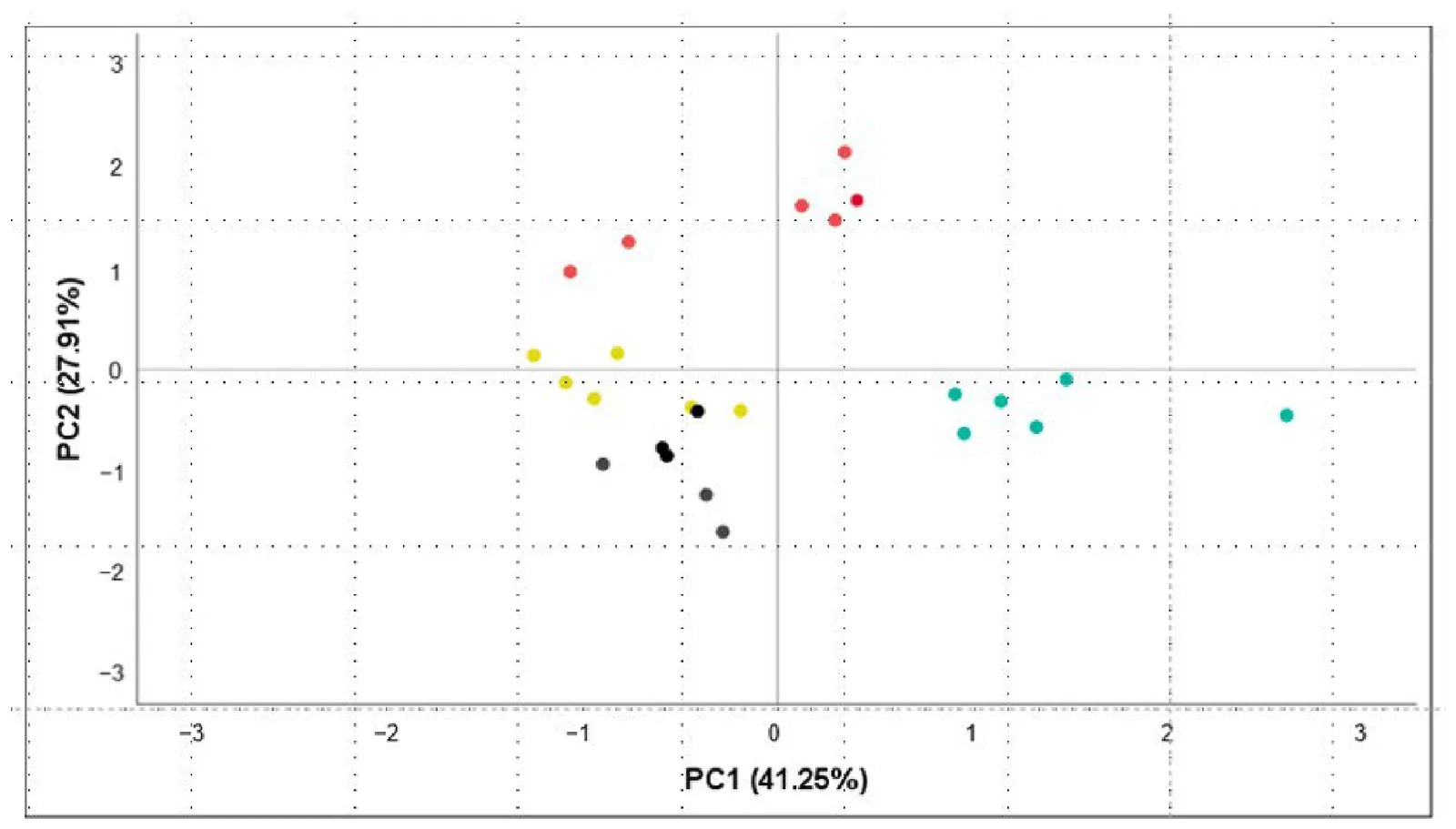
Principal component analysis of functional traits of *H. decurrens* plants grown in local natural soil (NS) and fire-disturbed soil (DS), without (NC) or with (WC) intraspecific competition. Each point represents one experimental pot (n = 6 per treatment). Symbols represent treatment combinations: yellow circles, NC-WC; black circles, DS-WC; green circles, DS-NC; red circles, NS-NC.

The PCA included nine functional traits (Figure 5). The KMO value was 0.635 and Bartlett’s test was significant (χ^2^ = 138.640, df = 36, *p* < 0.001), supporting the suitability of the data for PCA. The first two principal components explained 69.16% of the total variance (Component 1 = 41.25% and Component 2 = 27.91%). The first component was mainly associated with root length, proteoid root number, RMF and photosynthetic pigments (total chlorophyll and carotenoids), whereas the second component primarily contrasted shoot biomass and SS with phenolic compounds and photosynthetic pigments. The results also showed that the treatment combinations occupied distinct regions of the ordination space. Plants grown in fire-disturbed soil without competition (DS-NC) were predominantly positioned at positive PC1 scores, associated with greater belowground investment and pigment concentrations, whereas plants grown in local natural soil without competition (NS-NC) were primarily associated with positive PC2 scores and with shoot biomass and SS. Both competition treatments were displaced from their corresponding non-competition treatments, indicating marked changes in the overall functional profile under intraspecific competition.

## 4. Discussion

The results of the current study show that fire-disturbed soil did not consistently enhance juvenile growth of *H. decurrens*. However, soil condition induced significant changes in several morphological traits (shoot dry weight, root length, and proteoid roots), leaf biochemical traits (photosynthetic pigments, soluble sugars, and phenolic contents), nutritional traits (micronutrient contents), and easily extractable soil glomalin. These findings support the general view that soil factors influence biomass trade-offs by affecting plant resource availability [51]. Taken together, our findings only partially support the first hypothesis (H1). Contrary to the transient nutrient enrichment often associated with ash deposition immediately after fire [52], the fire-disturbed soil used in the experiment contained lower levels of organic matter (4.78% vs. 12.02%), total N (2.26 g kg^−1^ vs. 5.33 g kg^−1^) and extractable P (3 mg kg^−1^ vs. 6 mg kg^−1^) than the local natural soil. Post-fire changes in soil fertility are strongly time-dependent, as the initial increase in extractable nutrients associated with ash deposition can decline substantially within the first year after fire, partly through leaching and redistribution within the soil profile [2,3,53]. Therefore, the approximately one-year interval between the wildfire and soil collection may partially explain the relatively nutrient-poor conditions observed in the fire-disturbed soil in our study.

Despite nutrient depletion, plants grown in fire-disturbed soil maintained their overall growth and showed greater root length and proteoid root formation. Proteoid or cluster roots are dense clusters of short, determinate lateral rootlets that constitute a specialized nutrient-acquisition strategy found in several Proteaceae species [54]. These roots exude carboxylates and other compounds that mobilize poorly available nutrients, particularly P and several micronutrients [55]. Such adaptations are considered critical to the persistence of Proteaceae in severely P-impoverished soils [56]. In *Hakea prostrata*, for example, low P availability stimulates cluster-root formation, whereas an increased P supply suppresses investment in these structures [57]. Likewise, studies on invasive *Hakea* populations in Portugal, historically identified as *H. sericea* [23], demonstrated dense proteoid-root development in N- and P-poor soils, as well as a significant contribution of proteoid roots to phosphate acquisition under low-P conditions [56]. The higher production of proteoid roots in fire-disturbed soil, together with the maintenance of similar leaf P concentrations despite lower extractable soil P, is consistent with an adjustment in resource-acquisition strategy. This belowground adjustment is accompanied by higher concentrations of easily extractable glomalin in fire-disturbed soil, suggesting altered soil microbial dynamics that may interact with the plant’s nutrient-acquisition strategies. Overall, this suggests that adjustments in belowground resource acquisition may contribute to the ability of *H. decurrens* plants to maintain its juvenile performance in nutrient-poor soil conditions represented in our experiment.

Intraspecific competition exerted the most consistent effect on the individual performance of *H. decurrens*, fully supporting our second hypothesis (H2). Fire-triggered release from stored seed banks in the canopy can generate abundant and largely synchronous recruitment, as well as dense and even-aged cohorts in serotinous *Hakea* populations [30,58]. Under such conditions, increasing conspecific density inevitably intensifies competition for shared aboveground and belowground resources. In the present study, competition significantly reduced plant height, relative height growth rate, root length, shoot and root dry weight, stem diameter, and the number of proteoid roots. This demonstrates a broad constraint on individual growth rather than an effect restricted to a specific plant organ. The significant effect of competition on RGR_H_, together with the absence of a statistically significant time × competition interaction, also suggests that competition reduced growth consistently throughout the experiment without altering its temporal pattern. The reduction in individual biomass at high densities is a well-documented ecological trade-off, although its magnitude can depend strongly on resource availability and species characteristics [59].

Our results also reveal that the functional strategy of *H. decurrens* in response to competition is highly context-dependent, providing strong support for our third hypothesis of significant soil × competition interactions (H3). Depending on the soil conditions, competition had divergent effects on biomass allocation. The root mass fraction (RMF) remained almost unchanged under competition in local natural soil but decreased in fire-disturbed soil, while the reduction in shoot biomass was also dependent on soil condition. These significant soil × competition interactions indicate non-additive responses of both traits to soil condition and conspecific density. This context-dependent response has been reported across plant species, with nutrient availability modifying the effects of intraspecific competition on biomass allocation and functional morphology [59]. A comparable pattern has been observed in other woody species, such as *Quercus liaotungensis*, where the effects of intraspecific competition on biomass allocation are also mediated by soil conditions [60].

Leaf soluble sugars and starch also showed contrasting responses across the four soil × competition combinations, indicating a non-additive adjustment of carbon partitioning in *H. decurrens*. Competition reduced the concentration of soluble sugars but increased starch accumulation in plants grown in local natural soil, whereas the opposite pattern was observed in fire-disturbed soil, where starch declined and soluble sugars were maintained or slightly increased. These contrasting patterns indicate that the balance between available and stored carbon differed according to the combined soil and density environment, rather than reflecting a uniform response to either soil condition or competition alone. As soluble sugars and starch are dynamically interconnected pools involved in carbon transport, storage and source–sink regulation [61], the opposite changes observed in these fractions may reflect distinct carbon allocation patterns under competition, depending on soil conditions [62,63]. Rather than indicating a single metabolic response to increasing plant density, these results suggest that *H. decurrens* adjusts the balance between readily available and stored carbon according to the combined effects of soil condition and competition [64]. In parallel, phenolic content increased in fire-disturbed soil and under intraspecific competition, suggesting a shift in secondary metabolism that may be associated with chemical defense and tolerance [65]. Phenolic compounds participate in several protective and regulatory functions, and their accumulation frequently changes in response to environmental constraints. However, their accumulation alone cannot be considered direct evidence of physiological stress [66]. Plant–plant competition has also been shown to modify secondary metabolism [67], but the direction and magnitude of such responses are strongly species- and context-dependent.

Further analysis of leaf mineral composition showed that it was predominantly characterized by additive effects of soil condition or conspecific density, whereas Zn was the only element showing a significant soil × competition interaction. Fire-disturbed soil was associated with higher B, Fe and Cu but lower Mn concentrations, while competition reduced K concentrations and increased Fe and Mn concentrations. In contrast, Zn showed a non-additive response, with the highest concentration occurring in DS-WC plants. The cluster roots of Proteaceae can contribute to the mobilization and acquisition of poorly available micronutrients, including Zn, Mn and Cu, as demonstrated in *Hakea prostrata* [57]. However, because competition simultaneously reduced root growth and proteoid-root number in the present study, the high Zn concentration observed in fire-disturbed soil under competition cannot be directly attributed to greater proteoid-root development. Furthermore, as mineral concentrations rather than total nutrient contents were measured, possible biomass-related concentration or dilution effects cannot be excluded [68]. These complex interactions indicate that *H. decurrens* does not employ a fixed response to competition, but rather modulates its functional strategy according to the soil conditions, providing support for the third hypothesis (H3).

Taken together, our results suggest that fire-disturbed soil itself did not consistently favor the growth of juvenile *H. decurrens*. Nevertheless, plants maintained overall growth under the comparatively nutrient-poor conditions of the fire-disturbed soil through coordinated adjustments in belowground traits, carbon allocation, leaf biochemistry and mineral composition. Along with the high seedling emergence observed across soil types in our preliminary assay, these responses may facilitate the initial establishment of *H. decurrens* following disturbance. However, they should not be interpreted as direct evidence of successful establishment or of the formation of dense and persistent monospecific stands in natural post-fire environments. Such population-level outcomes depend on additional ecological and demographic processes, including seed production and dispersal, interactions with native vegetation, drought, herbivory, recurrent fire, and long-term population dynamics. From a management standpoint, our results highlight the importance of monitoring *H. decurrens* recruitment following fire, particularly during the early stages of population development.

## 5. Conclusions

The present study shows that fire-disturbed soil did not confer a generalized growth advantage to juvenile *H. decurrens*. Despite lower organic matter, N and extractable P in fire-disturbed soil, plants maintained overall growth while showing marked adjustments in root development, proteoid-root formation, leaf biochemistry and mineral composition. These responses highlight the ability of juvenile *H. decurrens* to adjust its resource-acquisition and carbon-allocation traits under nutrient-poor soil conditions. Such conditions alone may not constitute a strong limitation to early plant performance following disturbance. Intraspecific competition severely limits individual growth, while several responses to competition depended on soil condition, highlighting substantial plasticity during the juvenile stage.

The study was conducted on juvenile plants under experimental conditions over a 12-month period, and therefore does not directly address long-term establishment, population persistence or invasion dynamics under natural post-fire conditions. Further field studies across independent burned and unburned sites, and over longer post-fire periods, are needed to determine how plant functional responses, soil properties and population dynamics change through time.

## Figures and Tables

**Table 1:** Physicochemical properties of local natural soil (NS) and fire-disturbed (DS) soils used in the experiments.

Soil Characteristics	Local Natural Soil (NS)	Fire-Disturbed Soil (DS)
pH (H_2_O)/pH (KCl)	4.8/3.8	4.9/3.8
Electrical conductivity (dS m^−1^)	0.011	0.037
Organic matter (%)	12.02	4.78
C/N	13.1	12.3
CEC (cmolc kg^−1^)	3.37	3.56
Exchangeable Ca (cmolc kg^−1^)	1.77	1.86
Exchangeable Mg (cmolc kg^−1^)	0.54	0.55
Exchangeable K (cmolc kg^−1^)	0.09	0.10
Exchangeable Na (cmolc kg^−1^)	0.09	0.10
Exchangeable Al (cmolc kg^−1^)	0.88	0.96
Total N (g N kg^−1^)	5.33	2.26
Extractable P (mg P_2_O_5_ kg^−1^)	6	3
Extractable K (mg K_2_O kg^−1^)	45	39
Extractable micronutrients (mg kg^−1^)		
B	0.86	0.57
Cu	1.80	1.99
Zn	2.0	1.5
Fe	266.5	269.5
Mn	3.9	3.8

**Table 2:** Emergence-related parameters determined 90 days after sowing.

Parameter	Formula
Emergence percentage (G, %)	$G\;\left(\%\right)=\frac{\sum_{i=1}^{k}\mathrm{ni}}{N}\times 100$
Mean emergence time (MGT, day)	$\mathrm{MGT}=\frac{\sum_{i=1}^{k}\mathrm{ni}\times \mathrm{ti}}{\sum_{i=1}^{k}\mathrm{ni}}$
Coefficient of velocity of emergence (CVG, %)	$\mathrm{CVG}\;\left(\%\right)=\frac{\sum_{i=1}^{k}\mathrm{ni}}{\sum_{i=1}^{k}\mathrm{ni}\times \mathrm{ti}}\times 100$
Time to 50% emergence (T50, day)	$T50=\mathrm{ti}+\frac{\left(\frac{\sum_{i=1}^{k}\mathrm{Ne}}{2}-\mathrm{Ei}\right)\times (\mathrm{tj}-\mathrm{ti})}{\mathrm{Ej}-\mathrm{Ei}}$
Synchrony of emergence (Z)	$Z=\frac{\sum_{i=1}^{k}\mathrm{Cni},2}{C\sum \mathrm{ni},2}$ $,\;\mathrm{being}\;C=\frac{\mathrm{ni}(n1-1)}{2}$

ni—number of seeds emerged at observation time i; N—number of seeds sown; ti—time from sowing to observation i; k—last observation time. For T50 calculation, Ne is the final number of emerged seeds; Ei and Ej are the cumulative number of emerged seeds before and after Ne/2, respectively, and ti and tj are the corresponding observation times.

**Table 3:** Descriptive values of seedling emergence parameters (G, emergence percentage; MGT, mean emergence time; CVG, coefficient of velocity of emergence; T50, time to 50% emergence; Z, synchrony of emergence) in local natural (NS) and fire-disturbed soil (DS), under full light (FL), moderate light (ML) and low light (LL) conditions, after 90 days.

Parameter	NS-FL	NS-ML	NS-LL	DS-FL	DS-ML	DS-LL
G (%)	97.40	93.90	78.90	89.50	92.10	79.80
MGT (day)	66.80	60.85	62.13	66.56	64.88	63.92
CVG (%)	1.497	1.643	1.610	1.502	1.541	1.565
T50 (day)	61.54	60.35	61.88	60.88	63.50	63.20
Z	0.19	0.07	0.08	0.18	0.08	0.08

**Table 4:** Growth parameters, leaf biochemical parameters and leaf minerals of *H. decurrens* plants grown in local natural soil (NS) and fire-disturbed soil (DS), without (NC) or with (WC) intraspecific competition, at the end of the 12-month experiment. Values are means ± SE (n = 6). Different lowercase letters indicate significant differences among soil × competition interactions following post hoc multiple comparisons (*p* < 0.05).

Parameter	NS-NC	NS-WC	DS-NC	DS-WC	Soil (S)	Competition(C)	S × C
Growth parameters							
Height (cm)	32.2 ± 1.4	26.2 ± 1.6	31.3 ± 1.6	29.8 ± 0.9	n.s.	0.015	n.s.
Shoot DW (g)	14.2 ± 1.2 a	7.39 ± 0.45 c	10.9 ± 0.5 b	7.36 ± 0.27 c	0.033	<0.001	0.036
Root length (cm)	31.5 ± 1.5	25.9 ± 1.1	37.6 ± 3.1	27.1 ± 0.7	0.047	<0.001	n.s.
Root DW (g)	6.58 ± 1.12	3.34 ± 0.32	7.19 ± 0.46	2.82 ± 0.23	n.s.	<0.001	n.s.
Stem Ø (mm)	5.93 ± 0.31	4.89 ± 0.16	5.81 ± 0.11	4.72 ± 0.19	n.s.	<0.001	n.s
Branch number	41.7 ± 4.0	35.7 ± 3.0	43.7 ± 7.3	33.2 ± 2.8	n.s.	n.s.	n.s.
Proteoid roots	31.0 ± 4.3	19.6 ± 2.0	41.4 ± 2.7	21.3 ± 0.9	0.043	<0.001	n.s.
RMF	0.307 ± 0.032 ab	0.311 ± 0.022 ab	0.398 ± 0.012 a	0.271 ± 0.018 b	n.s.	0.016	0.011
Leaf biochemical parameters							
chl a (μg mg^−1^ FW)	0.569 ± 0.018	0.637 ± 0.051	1.36 ± 0.10	1.18 ± 0.08	<0.001	n.s.	n.s.
chl b (μg mg^−1^ FW)	0.303 ± 0.009 b	0.320 ± 0.026 b	0.725 ± 0.054 a	0.582 ± 0.042 a	<0.001	n.s.	0.046
chl total (μg mg^−1^ FW)	0.867 ± 0.027	0.952 ± 0.078	2.08 ± 0.15	1.75 ± 0.12	<0.001	n.s.	n.s.
chl a/chl b	1.88 ± 0.02	2.00 ± 0.07	1.88 ± 0.04	2.03 ± 0.04	n.s.	0.009	n.s.
carot (μg mg^−1^ FW)	0.146 ± 0.005	0.106 ± 0.006	0.200 ± 0.010	0.171 ± 0.010	<0.001	<0.001	n.s.
carot/chl total	0.168 ± 0.004 b	0.200 ± 0.009 a	0.162 ± 0.003 b	0.152 ± 0.003 b	<0.001	n.s.	0.001
Phenol (μg mg^−1^ FW)	21.1 ± 0.9	26.4 ± 0.7	24.5 ± 1.5	31.3 ± 0.8	<0.001	<0.001	n.s.
SS (μg mg^−1^ FW)	69.9 ± 2.6 a	53.4 ± 3.7 b	46.0 ± 3.3 b	50.5 ± 3.7 b	<0.001	n.s.	0.006
Starch (μg mg^−1^ FW)	32.2 ± 2.0 b	48.0 ± 3.0 a	50.9 ± 2.4 a	34.2 ± 2.2 b	n.s.	n.s.	<0.001
Leaf mineral contents							
P (g kg^−1^ DW)	0.440 ± 0.038	0.443 ± 0.019	0.457 ± 0.049	0.357 ± 0.020	n.s.	n.s.	n.s.
K (g kg^−1^ DW)	7.61 ± 0.37	7.02 ± 0.36	8.85 ± 0.79	6.86 ± 0.38	n.s.	0.020	n.s.
Ca (g kg^−1^ DW)	2.08 ± 0.15	2.17 ± 0.17	1.85 ± 0.05	2.21 ± 0.12	n.s.	n.s.	n.s.
Mg (g kg^−1^ DW)	1.37 ± 0.04	1.52 ± 0.05	1.37 ± 0.07	1.43 ± 0.09	n.s.	n.s.	n.s.
S (g kg^−1^ DW)	1.20 ± 0.06	1.23 ± 0.04	1.17 ± 0.08	1.12 ± 0.07	n.s.	n.s.	n.s.
B (mg kg^−1^ DW)	9.17 ± 0.65	8.83 ± 0.48	11.8 ± 0.9	10.2 ± 0.8	0.012	n.s.	n.s.
Fe (mg kg^−1^ DW)	143 ± 14	242 ± 13	290 ± 24	375 ± 27	<0.001	<0.001	n.s.
Zn (mg kg^−1^ DW)	30.9 ± 9.2 b	29.6 ± 3.9 b	34.8 ± 5.0 b	68.1 ± 9.7 a	0.013	n.s.	0.038
Mn (mg kg^−1^ DW)	127 ± 6	162 ± 9	103 ± 9	128 ± 11	0.003	0.003	n.s.
Cu (mg kg^−1^ DW)	3.67 ± 0.33	4.00 ± 0.26	5.50 ± 0.72	4.83 ± 0.48	0.012	n.s.	n.s.

## Data Availability

The data that support the findings of this study are available upon reasonable request from the corresponding author.

## Abbreviations

The following abbreviations are used in this manuscript:

NS	Local natural soil
DS	Fire-disturbed soil
NC	Absence of intraspecific competition
WC	Presence of intraspecific competition
